# Draft genome sequence and detailed characterization of biofuel production by oleaginous microalga *Scenedesmus quadricauda* LWG002611

**DOI:** 10.1186/s13068-018-1308-4

**Published:** 2018-11-09

**Authors:** Chitralekha Nag Dasgupta, Sanjeeva Nayaka, Kiran Toppo, Atul Kumar Singh, Uday Deshpande, Amitabikram Mohapatra

**Affiliations:** 1 Algology Laboratory, CSIR-National Botanical Research Institute, Rana Pratap Marg, Lucknow, Uttar Pradesh 226 001 India; 2 Bioserve|A CGI Company, 3-1-135/1A, CNR Complex, Mallapur, Hyderabad, Telangana 500 076 India

**Keywords:** Oleaginous microalgae, *Scenedesmus quadricauda*, Biofuel, Draft genome sequence, Lipid metabolism, Metabolic pathways, Phylogenetic analysis

## Abstract

**Background:**

Due to scarcity of fossil fuel, the importance of alternative energy sources is ever increasing. The oleaginous microalgae have demonstrated their potential as an alternative source of energy, but have not achieved commercialization owing to some biological and technical inefficiency. Modern methods of recombinant strain development for improved efficacy are suffering due to inadequate knowledge of genome and limited molecular tools available for their manipulation.

**Results:**

In the present study, microalga *Scenedesmus quadricauda* LWG002611 was selected as the preferred organism for lipid production as it contained high biomass (0.37 g L^−1^ day^−1^) and lipid (102 mg L^−1^ day^−1^), compared to other oleaginous algae examined in the present study as well as earlier reports. It possessed suitable biodiesel properties as per the range defined by the European biodiesel standard EN14214 and petro-diesel standard EN590:2013. To investigate the potential of *S. quadricauda* LWG002611 in details, the genome of the organism was assembled and annotated. This was the first genome sequencing and assembly of *S. quadricauda,* which predicted a genome size of 65.35 Mb with 13,514 genes identified by de novo and 16,739 genes identified by reference guided annotation. Comparative genomics revealed that it belongs to class Chlorophyceae and order Sphaeropleales. Further, small subunit ribosomal RNA gene (18S rRNA) sequencing was carried out to confirm its molecular identification. *S. quadricauda* LWG002611 exhibited higher number of genes related to major activities compared to other potential algae reported earlier with a total of 283 genes identified in lipid metabolism. Metabolic pathways were reconstructed and multiple gene homologs responsible for carbon fixation and triacylglycerol (TAG) biosynthesis pathway were identified to further improve this potential algal strain for biofuel production by metabolic engineering approaches.

**Conclusion:**

Here we present the first draft genome sequence, genetic characterization and comparative evaluation of *S. quadricauda* LWG002611 which exhibit high biomass as well as high lipid productivity. The knowledge of genome sequence, reconstructed metabolic pathways and identification of rate-limiting steps in TAG biosynthesis pathway will strengthen the development of molecular tools towards further improving this potentially one of the major algal strains for biofuel production.

**Electronic supplementary material:**

The online version of this article (10.1186/s13068-018-1308-4) contains supplementary material, which is available to authorized users.

## Background

The scarcity of fossil fuel as well as intricate link in climate change has necessitated the search for alternative fuel. In this context, oleaginous microalgae are in demand due to high biomass and lipid productivity as evidenced by accumulation of 40–80% TAG per gram dry weight [1].

Several algal strains including *Chlorella*, *Nannochloropsis*, *Scenedesmus, Kirchneriella* and *Selenastrum* have been screened for biomass and lipid productivity as they are easy to isolate, demonstrate growth robustness with high lipid content [2, 3]. *Scenedesmus* comprises entire desirable characteristics in one organism including high lipid production and wide growth range under diverse condition as compared to other oleaginous microalgae [4–10]. The lipid content of *Scenedesmus* varies from 10 to 50% of dry biomass according to strain and growth condition [4–7]. Out of all species, *S. quadricauda* is less exploited but could be an efficient candidate for future needs [5, 9, 10] as it produces ~ 50% lipid (weight/weight) of dry biomass [5] with desired quantity of saturated and unsaturated C16 and C18 fatty acids, which is a must for efficient biodiesel production [8].

In the present scenario, the maximum global microalgal biofuel productivity (when substrates are sufficient and conditions are within the optimal growth range) is estimated to be 3.2–14.8 TOE (tonnes of oil equivalent) ha^−1^ year^−1^ with a global average of 8.4 TOE ha^−1^ year^−1^ (in open ponds with 25% lipid content) [11]. An exceptionally high algal biofuel yield prediction of ~ 136.9 TOE ha^−1^ year^−1^ was reported by Chisti [1]; that is difficult to achieve in outdoor cultivation [12]. A wide range of prices from 1.3 to 20 USD L^−1^ have been reported for algal biofuel [13, 14]; thus demonstrating that algal biofuel is not yet cost competitive with petroleum based fuels or other biofuels [15]. However, scalability of the process is gearing up to meet sustainability [16].

The production of algal biofuel has several advantages such as utilizing nonfood-based feedstock, utilization of non-arable land for its cultivation and can potentially utilize a wide variety of water resources including wastewater and seawater [2, 3]. However, a major hurdle for this process is to achieve desired quantity of biomass and lipid content simultaneously. Most of the studies used nutrient deprivation and stress for culturing algae to induce more lipid production, but end up reducing the growth rate, thus affecting biomass productivity [8].

The identification of super productive strain is one potential solution which concomitantly with targeted metabolic engineering approaches can be applied to enhance the quantity of desired fatty acids to improve biodiesel quality, thus overcoming biological inefficiency of strain [8, 17–23]. However, recombinant strain development enhancing biofuel production is suffering due to lack of adequate knowledge of its genome and limited molecular manipulating tools [17–23]. One of the best examples is *Chlamydomonas reinhardtii* which has undergone extensive genetic manipulation because of availability of its genome sequence [20, 21]. In this context, genome of *Nannochloropsis oceanica*, *Nannochloropsis gaditana*, *Monoraphidium neglectum* and *Tetradesmus obliquus* were sequenced to identify the lipid biosynthetic pathways and their suitability for biofuel production [17, 18, 22, 23]. However, neither of these algae is found to be an exceptional producer of biomass or lipid. With few exceptions, average microalgal biomass productivity ranges from 0.02 to 0.40 g L^−1^ day^−1^ and lipid productivity ranges from 5 to 178 mg L^−1^ day^−1^. None of the algae has been commercialized for biodiesel production despite years of extensive efforts in research [15].

In the present study, we have investigated *S. quadricauda* LWG002611as a potential strain for biomass and lipid production. Furthermore, we have genetically characterized the strain followed by draft assembly for comparative genomics, identified functional genes and reconstructed important metabolic pathways. This is the first report on genome sequencing of *S. quadricauda* LWG002611. Based on the sequence, we have identified gene homologs of key enzymes involved in carbon fixation and TAG biosynthesis pathways, which can be targeted for genetic and metabolic engineering to further improvement of the strain.

## Results and discussion

### Selection of *S. quadricauda* LWG002611 as preferred organism for biodiesel production

**Fig. 1:**
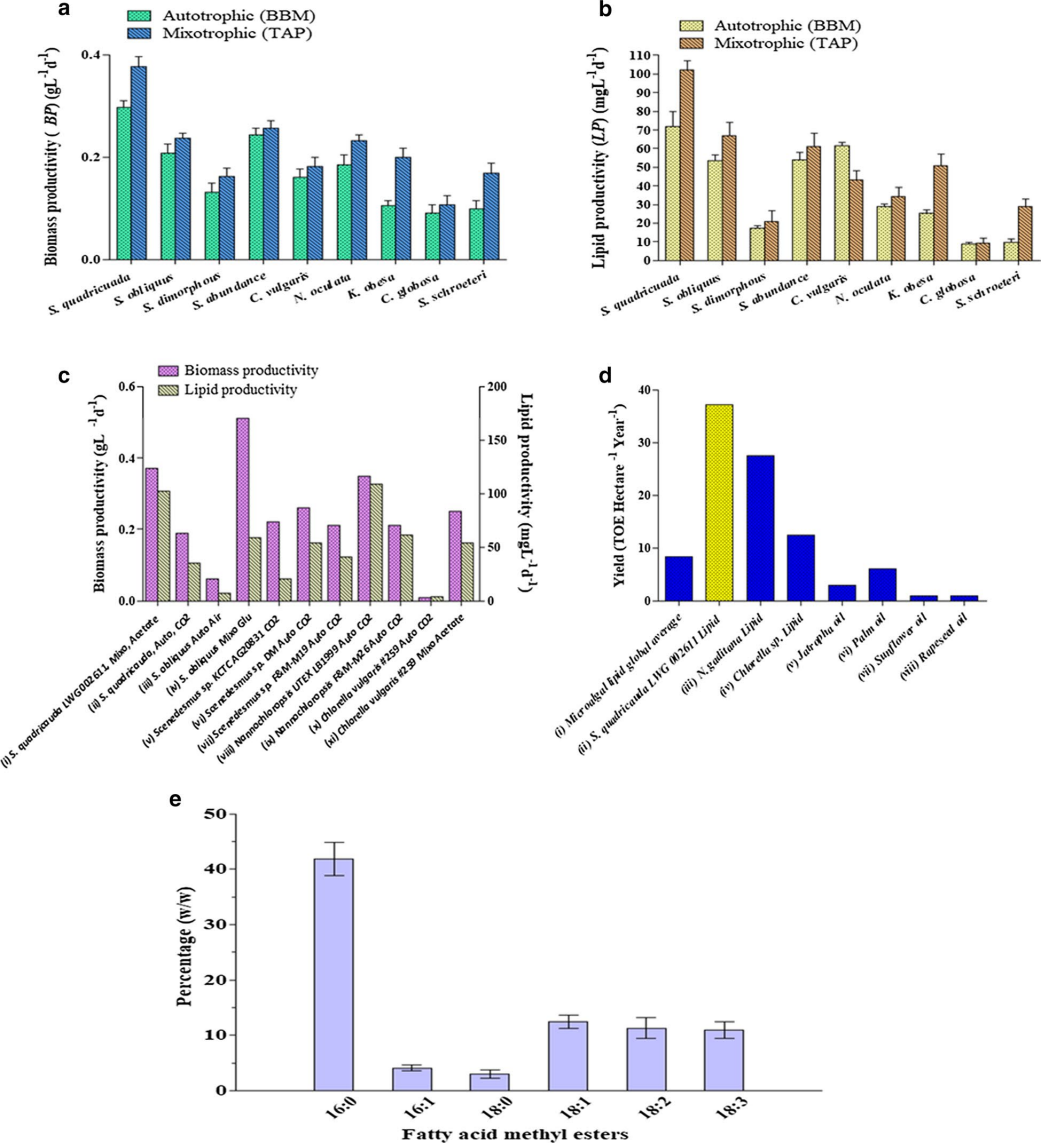
Comparison of **a** biomass productivity (g L^−1^ day^−1^) and **b** lipid productivity (mg L^−1^ day^−1^) of *S. quadricauda* LWG002611 with other eight isolated oleaginous microalgae grown in autotrophic cultivation condition using BBM and mixotrophic cultivation condition using TAP media where acetate is supplemented as external carbon source in uniform cultivation condition at a temperature of 27 °C ± 0.5 °C, a photoperiod of 14:10 h light/dark cycle and fluorescent illumination of 3000 lux. Productivity was estimated in logarithmic phase of growth. **c** Comparison of biomass productivity (g L^−1^ day^−1^) and lipid productivity (mg L^−1^ day^−1^) of *S. quadricauda* LWG002611 with earlier reports of microalgae grown in different carbon sources i.e. mixotrophic (mixo) growth in acetate and autotrophic (auto) growth in CO_2_ and air. Bar (i) indicate our estimation, bar (ii, vi, vii, ix) indicate the estimation by Rodolfi et al. [9], bar (iii, iv) by Mandal and Mallick [26], bar (v) by Yoo et al. [27], bar (viii) by Chiu et al. [28] and bar (x, xi) by Liang et al. [29]. **d** Comparison of *S. quadricauda* LWG002611 large-scale production rates (TOE ha^−1^ year^−1^) with other promising oleaginous microalgae as well as with other biofuel feedstocks. Bar (i) indicate the average global productivity of microalgal lipid [11], bar (ii) indicate our estimations for *S. quadricauda* LWG002611, extrapolated from 1 L cultures cultivated in TAP medium at a temperature of 27 °C ± 0.5 °C, a photoperiod of 14:10 h light/dark cycle and fluorescent illumination of 3000 lux; bar (iii) indicate extrapolated estimations of lipid productivity of *Nannochloropsis gaditana* in nitrogen deficient condition [18], bar (iv) indicate the lipid productivity of *Chlorella* sp. [18], bar (v), (vi), (vii) and (viii) represent the oil productivity of Jatropha [18], Palm [1], Sunflower [1] and Rapeseed [1] respectively. **e** The extracted lipid (mg) from biomass (g) of *S. quadricauda* LWG002611 was refluxed for 5 h at 50 °C in the presence of methanol and 2% sulphuric acid for transesterification. After removal of impurities the FAME mix (mg mg^−1^ of lipid) was dissolved in hexane and estimated for the percentage of Fatty acid methyl ester (FAME) (weight/weight) by Gas-Chromatography (Thermo Fisher Scientific) and quantified against a standard FAME mix (Supelco, USA) (values are from three separate experiments and error bars show the standard deviation)

*S. quadricauda* LWG002611 was found to be the most efficient strain for biomass and lipid production under both autotrophic and mixotrophic growth condition when compared to other species of *Scenedesmus* (*S. obliquus*, *S. dimorphus*, *S. abundans*) as well as other oleaginous microalgal isolates (*Chlorella vulgaris, Nannochloropsis oculata, Kirchneriella obesa, Chlamydomonas globosa*, *Sphaerocystis schroeteri*). It grew faster in mixotrophic growth condition (supplemented with 17.4 mM acetate in TAP medium) than autotrophic condition (in BBM) and achieved stationary growth phase within 8 days of culture. The maximum biomass productivity (0.37 g L^−1^ day^−1^) achieved by *S. quadricauda* in TAP medium; which was 27.5% more than the biomass productivity (0.29  g L^−1^ day^−1^) achieved in BBM in exponential phase of growth (Fig. 1a). After 25 days of growth, biomass of all isolates were harvested and lyophilized and total biomass yield was estimated. *Scenedesmus, Nannochloropsis* and *Chlorella* were found to be the highest biomass yielding isolates. However, the maximum biomass content of 1.41 g L^−1^ was achieved by *S. quadricauda* LWG002611 followed by 1.29 g L^−1^ achieved by *N. oculata* in TAP medium. As expected, highest lipid productivity was observed in *S. quadricauda* (102 mg L^−1^ day^−1^) in TAP media followed by *S. obliquus* (67 mg L^−1^ day^−1^) without any starvation (Fig. 1b). The total lipid content of 404 mg L^−1^ was achieved by *S. quadricauda* in TAP medium without nitrogen deprivation or any other starvation. This study mirrors what has been found earlier that additions of organic carbon (acetate) boosted up total carbon assimilation of algae and helped to improve biomass as well as lipid production [7]. It produced 1.9-fold more biomass and 2.9-fold more lipid than the *S. quadricauda* as screened by Rodolfi et al. [9]. Even though nutrient starvation has been reported to be the most suitable stimulant to raise lipid content [4–9, 18, 22], it is difficult to maintain nutrient deficient environment for large scale cultivation in open ponds. Moreover, in nutrient starvation model, although the lipid content increases, but it significantly reduces algal growth thus, affecting biomass content [8]. Thereby, we have aimed to identify high lipid yielding algae without any starvation and stress.

In general biomass productivity ranges from 0.02 to 0.40 g L^−1^ day^−1^ and lipid productivity ranges from 5 to 178 mg L^−1^ day^−1^ [24]. As compared, *S. quadricauda* LWG002611 (biomass productivity 0.37 g L^−1^ day^−1^ and lipid productivity 102 mg L^−1^ day^−1^) is on the higher side of the range (Fig. 1c) [9, 25–29]. Under large scale production, many studies have estimated the maximum productivity of algal biofuels ranges from 8.2 to 136.9 TOE ha^−1^ year^−1^ [30]. In some cases, the data from lab scale experiments are used to estimate the productivity of large scale production whereas some are real large scale production values. Our laboratory productivity numbers of *S. quadricauda* LWG002611 have been extrapolated and found to be on higher side compared to other large-scale biofuel production platforms and are higher than the global average productivity (Fig. 1d) [1, 11, 18].

### Fatty acid profile and biodiesel properties of *S. quadricauda* LWG002611

The fatty acid profile of *S. quadricauda* LWG002611 was found to be simple as compared to other known oleaginous algae, as it contains only six fatty acids (FA) of carbon chain length C16–C18 such as palmitic acid (16:0) 44.46%, oleic acid (18:1) 12.37%, linoleic acid (18:2) 11.29% and linolenic acid (18:3) 10.95% (Fig. 1d). According to EN14214, the percentage of linolenic acid (C18:3) and highly polyunsaturated FAs (≥ 4 double bond) in the biodiesel should not exceed the maximum limit of 12% and 1%, respectively [7, 31, 32]. In the present study, the percentage of linolenic acid was found to be 10.9% while polyunsaturated FAs with ≥ 4 double bond were completely absent, thus making it desirable for biodiesel production (Fig. 1d). The fatty acid methyl ester (FAME) profile of *S. quadricauda* LWG002611 was compared with other two oleaginous algae such as *N. gaditana* CCMP526 [18] and *M. neglectum* [22] whose genome was known and already been characterized for biofuel production. *N. gaditana* CCMP526, the high abundance of palmitic acid was observed along with an abundance of myristic acid (C14:0) as well as C16 unsaturated fatty acids, which were not detected in *S. quadricauda* LWG002611 [18]. In comparison, *M. neglectum* showed the high abundance of oleic acid (C18:1) and low abundance of saturated acids [22].

The biodiesel properties of FAME of *S. quadricauda* LWG002611 were found to be within the limits of European standard EN14214 and EN 590:2013 (Table 1) [33, 34]. The FAMEs of *S. quadricauda* exhibits very less unsaturation and all the indices related to unsaturation such as degree of unsaturation (DU), iodine value (IV), allylic position equivalent (APE) and the bisallylic position equivalent (BAPE) were found to be within the range of EN14214 and EN 590:2013 [33, 34] (Table 1). The combustion quality indices cetane number (CN), higher heating value (HHV) and saponification value were also within the limits [35] (Table 1). Another bottleneck of biofuel is inferior cold flow properties characterized by cloud point (CP), pour point (PP), cold filter plugging point (CFPP) and viscosity (*υ*) [36] (Table 1). Use of a fuel in colder regions depends upon its cold flow properties. Cold flow properties of FAMEs obtained were well within the accepted range of standard fuels. The CP and PP was obtained as 17 °C and 11.7 °C respectively that indicates biodiesel obtained from *S. quadricauda* LWG002611 will become cloudy and semisolid at the respective temperatures. Furthermore, cold filter plugging point (CFPP), the most important parameter for cold flow properties was obtained as 1.3 °C, which indicates the lowest temperature for free flowing fuel systems. The kinetic viscosity value (*υ*) [37] and fuel density (*ρ*) are also strongly influenced by temperature [38] (Table 1).

**Table 1:** Comparison of biodiesel properties of *S. quadricauda* LWG002611 with standards of biodiesel and petro-diesel

Biodiesel properties	*S. quadricauda* LWG002611 biodiesel	Biodiesel specifications (EN14214)	Petro-diesel (EN 590:2013)
Saturated fatty acid (SFA) (%)	44.825	–	–
Mono unsaturated fatty acid (MUFA) (%)	16.461	–	–
Poly unsaturated fatty acid (PUFA) (%)	22.250	–	–
Degree of unsaturation (DU)	60.961	< 137	–
Saponification value (SV) (mg g^−1^)	175.371	–	–
Iodine value (IV)	65.651	< 120	–
Cetane number (CN)	62.651	> 51	> 51
Long chain saturated factor (LCSF)	5.669	–	–
Cold filter plugging point (CFPP) (°C)	1.334	Varies	− 5 to − 15 °C
Cloud point (CP) (°C)	17.025	Varies	–
Pour point (PP) (°C)	11.661	Varies	–
Allylic position equivalent (APE)	56.876	–	–
Bis-allylic position equivalent (BAPE)	33.208	–	–
Oxidation stability (OS) (g m^−3^)	7.891	> 6	< 25
Higher heating value (HHV) (MJ kg^−1^)	32.816	–	44.8
Kinematic viscosity (*υ*) (mm^2^ s^−1^) at 40 °C	2.937	3.5–5.0	2.0–4.5
Density (*ρ*) (kg m^−3^) at 15 °C	731	860–900	820–845

### Genome sequencing, draft assembly, functional annotation and comparative genomics of *S. quadricauda* LWG002611

Although, *S. quadricauda* LWG002611 is a high biomass and lipid yielding microalgae, the production level is still a crucial criteria to make it possible candidate for economically sustainable biofuel production [15]. Thus molecular and genetic modifications to improve fatty acid contents or yield of *S. quadricauda* LWG002611 are desired.

We are reporting for the first time whole genome sequencing of *S. quadricauda* LWG002611 followed by de novo and reference assisted assembly with currently available tools and databases (Additional files 1, 2, 3). Genome sequencing was performed using Ion Torrent Next-Generation Sequencing Technology (NGS) where a total of 57.27 million reads were obtained for further analysis (Additional file 1). A complete set of 13,514 functional genes were identified from de novo assembly (Table 2). After cleaning, the reads were processed for reference mapping with *M. neglectum* (NCBI accession no NW_014013625.1 with genome size of 69.71 Mb), a closely related species of Chlorophyceae. 83.85% of the reads mapped to reference and the genome size recovered was 65.35 Mb, indicating a > 90% genomic coverage. Further, to identify gene similarity with other algae, the annotated genes were processed for gene ontology in UniprotKB with 16,739 genes identified [22]. Reference-guided assembly has been reviewed in NCBI BioProject and assigned accession number NNCB00000000. The statistics pertaining to bioinformatics assembly are tabulated in Table 2.

**Table 2:** De novo genome assembly and reference-guided assembly statistics of *S. quadricauda* LWG002611

Statistics of genome assembly							
Estimated genome size 65.35 Mb							
De novo assembly		Reference-guided assembly		Misassemblies		Mismatches	
Total reads (counts)	57,273,557	Largest alignment (bp)	55,459	Misassemblies	2	Mismatches	127,490
Matched (counts)	53,419,545	Total aligned length (bp)	6,376,645	Relocations	2	Indels	95,642
Mismatched (counts)	3,854,012	Contigs	13,425	Translocations	0	Indels length	102,952
Contigs (counts)	58,317	Contigs (≥ 0 bp)	14,013	Inversions	0	Mismatches per 100 kbp	1982.82
Contig maximum length (bp)	368,038	Contigs (≥ 1000 bp)	11,309	Misassembled contigs	2	Indels per 100 kbp	1487.5
Contig minimum length (bp)	358	Contigs (≥ 5000 bp)	4344	Misassembled contigs length (bp)	12,585	Indels (≤ 5 bp)	95,627
Contig average length (bp)	1704	Contigs (≥ 10,000 bp)	1653	Local misassemblies	216	Indels (> 5 bp)	15
N75	996	Contigs (≥ 25,000 bp)	150	Unaligned miscontigs	2535	N’s	0
N50	2112	Contigs (≥ 50,000 bp)	3			N’s per 100 kbp	0
N25	16,976	Largest contig (bp)	68,181				
Total bases	99,381,020	Total length (bp)	65,171,243				
GC%	32.5	Total length (≥ 0 bp)	65,393,757				
Genes	13,514	Total length (≥ 1000 bp)	63,962,748				
		Total length (≥ 5000 bp)	45,404,946				
		Total length (≥ 10,000 bp)	26,423,640				
		Total length (≥ 25,000 bp)	4,731,985				
		Total length (≥ 50,000 bp)	180,197				
		N50	8094				
		N75	4282				
		L50	2350				
		L75	5093				
		GC (%)	63.2				
		Genes	16,739				

This assembly and mapping statistics were generated by Quality Assessment Tool for Genome Assemblies’ (QUAST). The de novo assembly and the reference mapping the filtered reads were processed in CLC bio Genomic Workbench 9.0 de novo assembly and reference mapping tool

The unavailability of the genomic sequence of *S. quadricauda* hampers the development of a more commercially viable strain, thus this attempt to elucidate its whole genome. The genome is currently in “draft assembly” stage as all the genes of its genome could not be identified by currently available tools and data bases. Based on the de novo and reference-assisted approach, although more than 90% of its genome information could be identified, still there are gaps in the assembly. One of the main reasons behind was the unavailability of closely related algal genome data to compare and authenticate the assembly. With the advancement of genome sequencing tools and platforms as well as continuous availability of novel genome information, all the gaps will be identified in future.

**Fig. 2:**
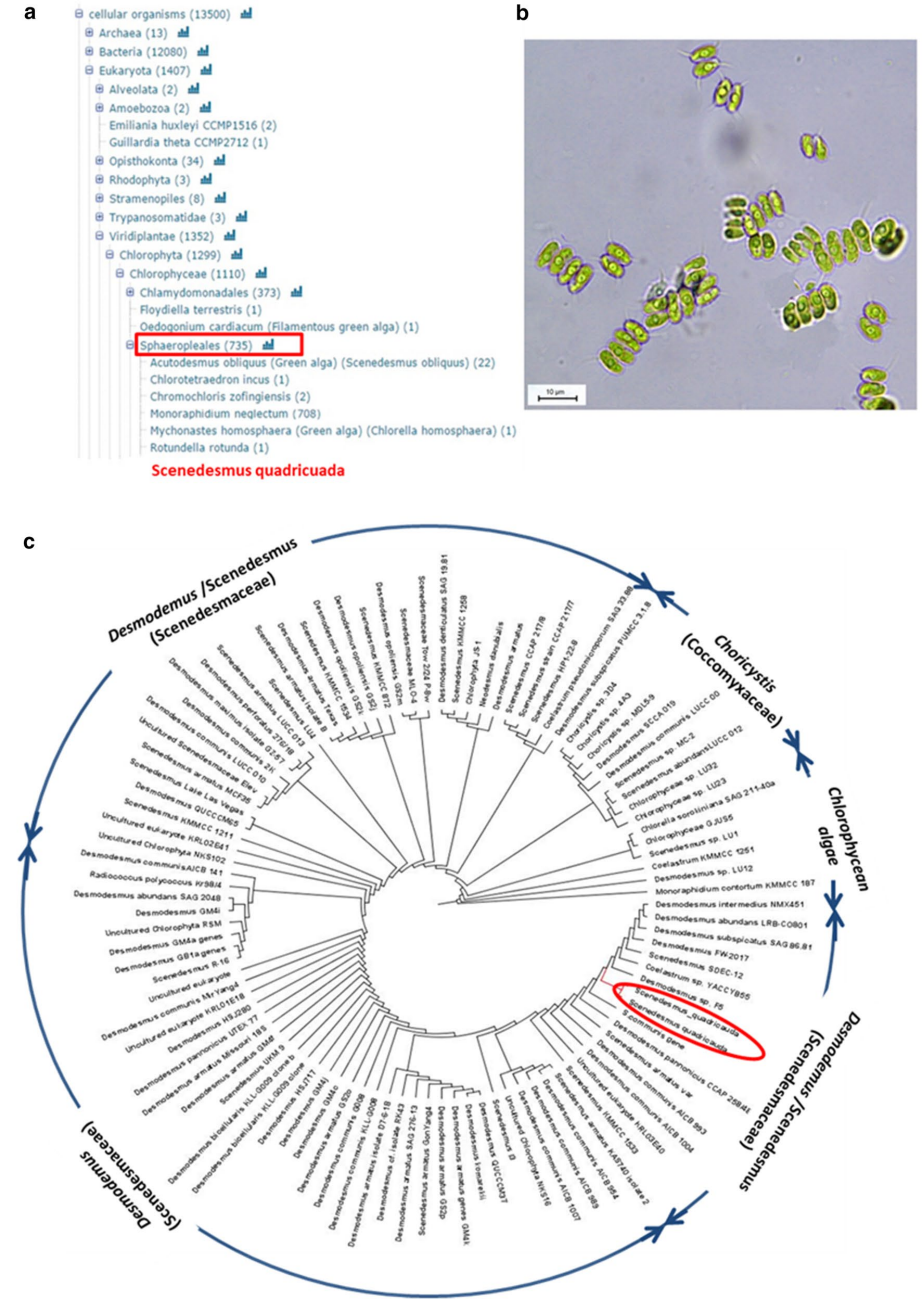
**a** Phylogenetic tree constructed from de novo genome sequence annotation using Uniprot hits from Cellular component of the algae families and species. **b** Photomicrograph of *S. quadricauda* LWG002611 under light microscope (×63). **c** Phylogenetic relationship of *S. quadricauda* LWG002611 with other algae based on 18S rDNA sequences using NR database of NCBI blast and figure was generated by Mega 5 software

Based on annotation from de novo assembly, *S. quadricauda* LWG002611 belongs to empire Eukaryota, sub-kingdom Viridiplantae, phylum Chlorophyta, class Chlorophyceae and order Sphaeropleales (Fig. 2a). To resolve the anomaly, small subunit ribosomal RNA marker gene (18S rRNA) based identification was carried to identify the organism. The sequence of 18S rRNA gene was submitted to NCBI gene bank (accession no. KY654954), which demonstrated 99% similarity with partial sequence of *Desmodesmus intermedius* strain NMX451 18S rRNA gene (Fig. 2c). The holotype species of *Desmodesmus* is *Scenedesmus quadricauda* (Turpin) Brébisson (Algaebase) [39]. A phylogenetic relationship was drawn on the basis of 18S rDNA sequence and NCBI blast. It was mostly clustered with different strains of *Desmodesmus* and *Scenedesmus* (Fig. 2c). Further, morphological characterization was carried out to confirm the identification of the organism (Fig. 2b) [40, 41]. The sample of *S. quadricauda* is preserved in CSIR-National Botanical Research Institute herbarium with accession number LWG002611 and germplasm is being maintained. The insufficient availability of DNA sequencing data is one of the major bottlenecks of DNA based taxonomy [42], thus further validation through morphological analysis is required [43].

### Comparative analysis of predicted gene function and reconstruction of bioenergy metabolic pathways

**Fig. 3:**
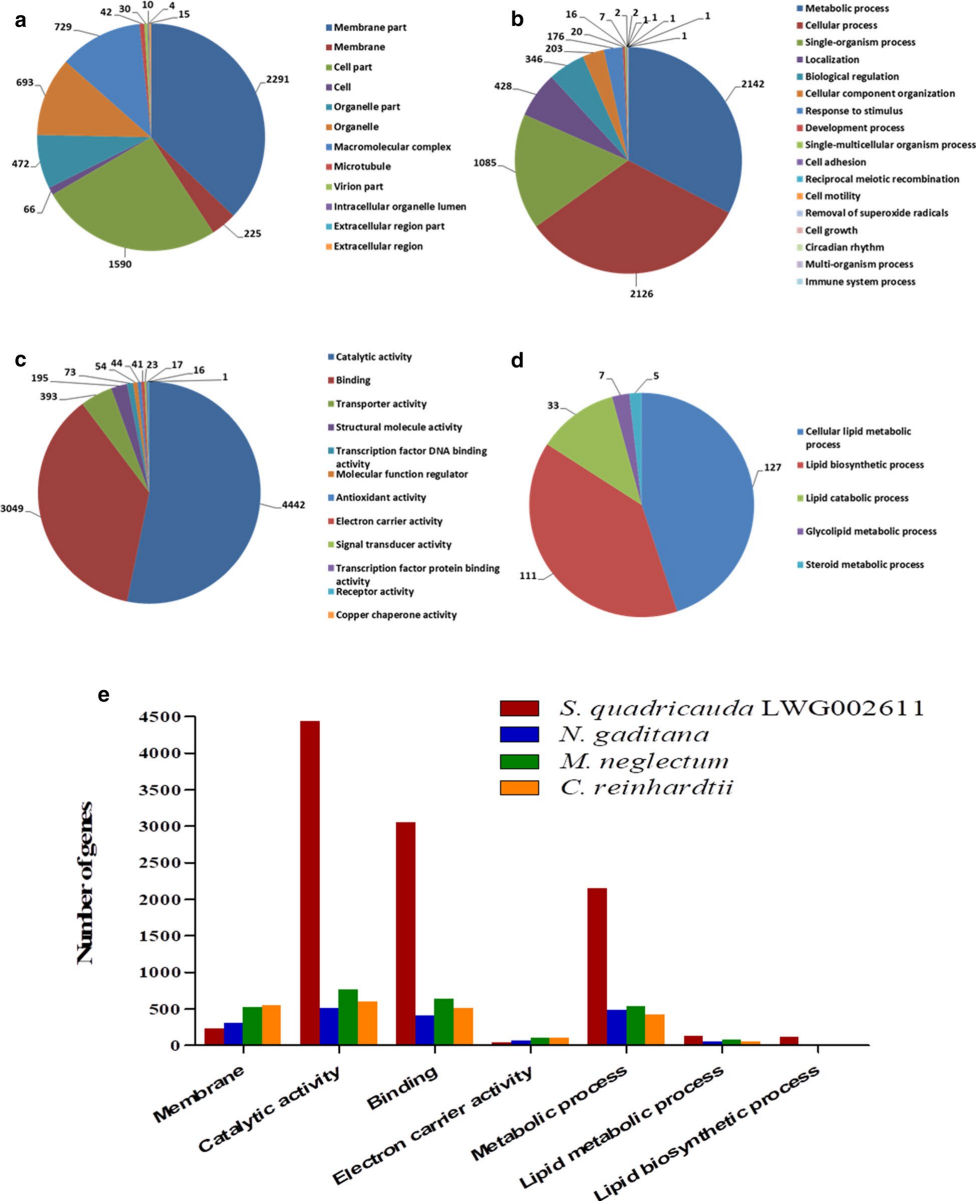
The above figures represent the gene ontology (GOs) of *S. quadricauda* LWG002611 in Panther gene ontology. **a** The number of genes involved in cellular component. **b** The number of genes involved in biological process. **c** The number of genes involved in molecular function. **d** The number of genes involved in lipid metabolism. **e** Comparative gene numbers related to membrane, catalytic activity, binding, electron carrier activity, metabolic process, lipid metabolic process, lipid biosynthetic process of *S. quadricauda* LWG002611, *N. gaditana, M. neglectum* and *C. reinhardtii* (the gene ontology of *S. quadricauda* LWG002611 is given in Additional file 5) [18, 21, 22]

Investigation of metabolic pathways of interest and identification of bottlenecks for production are the major concerns of this work. The majority of *S. quadricauda* genes were present in catalytic activity, binding, metabolic processes, lipid metabolism and lipid biosynthetic processes. A total of 6167 genes were identified for cellular component in gene ontology terms (GOs), 6558 for biological processes, 8348 for molecular functions and 283 for lipid metabolism, amongst them some genes are common in all processes (Fig. 3a–d, Additional files 4, 5). Furthermore, special emphasis was laid on lipid metabolic pathway genes that are involved in lipid biosynthesis, TAG assembly and lipid catabolism (Fig. 3d). The genes involved in lipid metabolism were categorized in cellular lipid metabolic process and consisted of 127 genes, 111 genes were identified for lipid biosynthesis process and 33 genes were identified in lipid catabolism (Fig. 3d). Similarly, for glycolipid metabolism seven genes were identified while only 5 were identified for steroid metabolic process (Fig. 3d). The total gene numbers of *S. quadricauda* LWG002611 was compared with *N. gaditana, M. neglectum* and *C. reinhardtii* (Fig. 3e). It demonstrated larger number of genes annotated for overall catalytic activity (4442), binding activity (3049), overall metabolic process (2142), lipid metabolic process (127) and lipid biosynthetic process (111) (The Gene ontology of *S. quadricauda* LWG002611 is given in Additional file 5). Genome sizes of *N. gaditana*, *M. neglectum* and *C. reinhardtii* were reported as 29 Mb, 69.71 Mb and 120 Mb encoding 9052, 16,761, 17,743 protein coding genes respectively [18, 21, 22]. This higher abundance of genes in *S. quadricauda* LWG002611 is reflecting in a larger regulatory and metabolic repertoire which could lead to high lipid accumulation in *S. quadricauda*. However, detailed functional investigation and transcriptome mapping are required to confirm the expression of gene families and to eliminate the pseudogenes.

Photosynthesis is an essential process to harness light energy for metabolism. Several genes that encoded components of Photosystem II, Photosystem I, Cytochrome b6/f complex and electron transport chain were identified from annotated genome sequence of *S. quadricauda* LWG002611. For Photosystem II, genes for photosystem b (Psb) components Psb O, Psb P, Psb Q, Psb R, Psb S and Psb 27 were identified. For Photosystem I, genes for photosystem a (Psa) components Psa E, Psa F, Psa G and Psa O were identified. Genes for photosynthetic electron transport (Pet) components Pet B, Pet C, Pet E, Pet F and Pet H were also identified. Genes for metal detoxification were also identified.

**Fig. 4:**
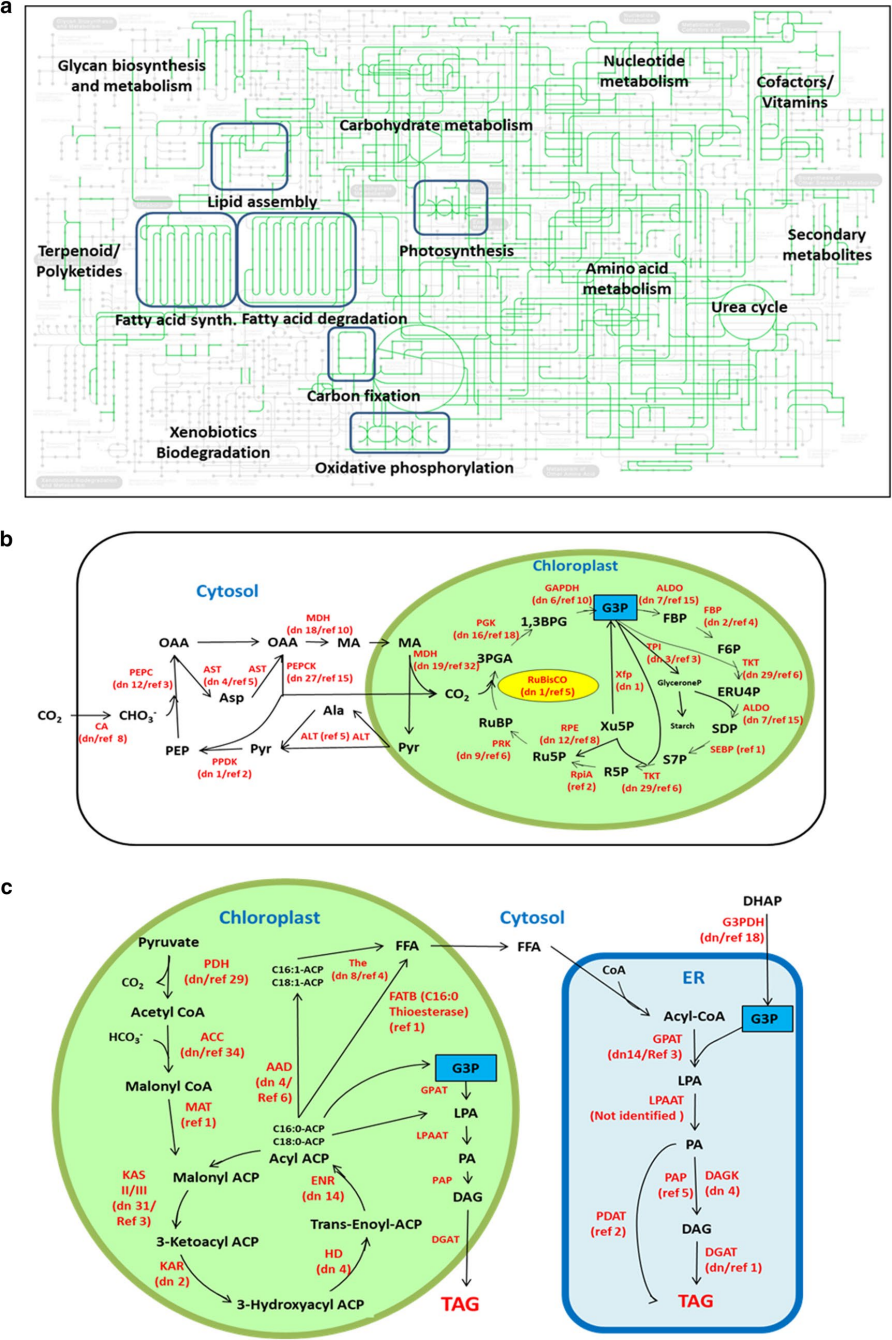
**a** Reconstructed metabolic pathways (KEGG) of *S. quadricauda* LWG002611. The pathways encoded by it are represented by green colour and not encoded are light grey in colour. **b** Reconstructed carbon fixation pathway showing the number of homologous genes found for the respective enzyme (number of the homologous gene given in the first bracket) in genome sequence of *S. quadricauda* LWG002611: PEP, phosphoenolpyruvate; OAA, oxaloacetate; MA, malic acid; Pyr, pyruvate; 3PGA, 3-phosphoglycerate; 1,3BPG, 1,3-bisphosphoglycerate; G3P, glyceraldehyde 3-phosphate; FBP, fructose 1,6-bisphosphatase; F6P, Fructose 6-phosphate; ERU4P, Erythrose 4-phosphate; SDP, Sedoheptulose-1,7-bisphosphatase; S7P, sedoheptulose 7-phosphate; R5P, ribose 5-phosphate; Ru5P, ribulose 5-phosphate; RuBP, ribulose-1,5-bisphosphate; enzymes (in red): PEPC, phosphoenolpyruvate carboxylase [EC:4.1.1.31]; MDH, malate dehydrogenase [EC:1.1.1.82]; PEPCK, Phosphoenolpyruvate carboxykinase [EC:4.1.1.49]; PPDK, pyruvate, orthophosphate dikinase [EC:2.7.9.1]; AST, aspartate aminotransferase [EC:2.6.1.1]; ALT, alanine transaminase [EC:2.6.1.2]; PGK, phosphoglycerate kinase [EC:2.7.2.3]; GAPDH, glyceraldehyde 3-phosphate dehydrogenase [EC:1.2.1.12]; ALDO, fructose-bisphosphate aldolase, class I [EC:4.1.2.13]; FBP, fructose-1,6-bisphosphatase I [EC:3.1.3.11]; TKT, transketolase [EC:2.2.1.1]; SEBP, sedoheptulose-1,7-bisphosphatase [EC: 3.1.3.37], RpiA, ribose 5-phosphate isomerase A [EC:5.3.1.6]; PRK, phosphoribulokinase [EC:2.7.1.19]; TPI, triosephosphate isomerase [EC:5.3.1.1]; Xfp, xylulose-5-phosphate [EC:4.1.2.9]; RPE, ribulose-phosphate 3-epimerase [EC:5.1.3.1]; CA, carbonic anhydrase [EC 4.2.1.1]; dn, de novo; ref, reference guided. **c** Reconstructed fatty acids and triacylglycerols (TAG) biosynthesis pathway showing the homologous gene found for the respective enzyme (number of the homologous gene given in the first bracket) in genome sequence of *S. quadricauda* LWG002611: FFA, free fatty acid; LPA, lysophosphatidic acid; PA, phosphatidic acid; DAG, diacylglycerol; TAG, triacylglycerol; ACP, acyl carrier protein; CoA, coenzyme A; enzymes (in red): PDH, pyruvate dehydrogenase complex [EC 1.2.4.1]; ACC, acetyl-CoA carboxylase [EC 6.4.1.2]; MAT, malonyl-CoA:ACP transacylase [EC 2.3.1.39]; KAS, β-ketoacyl-ACP synthase [EC 2.3.1.41]; KAR, β-ketoacyl-ACP reductase [EC 1.1.1.100]; HD, 3-hydroxyacyl-ACP dehydratase [EC 4.2.1.59]; ENR, enoyl-ACP reductase [EC 1.3.1.9]; FAT, fatty acyl-ACP thioesterase [EC 3.1.2.14]; G3PDH, gycerol-3-phosphate dehydrogenase [EC 1.1.1.8]; GPAT, glycerol-3-phosphate acyltransferase [EC 2.3.1.15]; LPAAT, lyso-phosphatidic acid acyltransferase [EC 2.3.1.51]; DAGK Diacylglycerol kinase [2.7.1.107]; PAP, Phosphatidic acid phosphatase [EC 3.1.3.4]; PDAT, phospholipid:diacylglycerol acyltransferase [EC 2.3.1.158]; DGAT, diacylglycerol acyltransferase [EC 2.3.1.20]; dn, de novo; ref, reference guided

Metabolic pathways of *S. quadricauda* LWG002611 were reconstructed using annotated genome sequence. The completeness of reconstructed pathways indicated that the gene function assignments were biologically meaningful. Metabolic pathways associated with biosynthesis and catabolism of lipid, carbohydrate, protein and nucleic acids are highlighted in Fig. 4; whereas metabolic pathways related to N-glycan synthesis, xenobiotics biodegradation, cofactors and vitamin biosynthesis as well as secondary metabolites biosynthesis were either incomplete or absent (Fig. 4a).

Microalgae are employed as carbon concentrating methods (CCMs) to increase intercellular carbon concentration. We have identified eight homologous genes for carbonic anhydrase (CA) which is responsible for conversion of atmospheric CO_2_ to HCO_3_^−^ soluble in cytosol (Fig. 4b). We also identified the large gene numbers that are required for C4 like mechanism such as phosphoenolpyruvate carboxylase (PEPC) (de novo assembly 12, reference-guided assembly 3), malate dehydrogenase (MDH) (de novo assembly 19, reference-guided assembly 32), phosphoenolpyruvate carboxykinase (PEPCK) (de novo assembly 27, reference-guided assembly 15), which helps the strain to assimilate carbon in variety of ecological niches (Fig. 4b). High abundance of genes of carbon assimilation is probably the reason for high biomass accumulation in *S. quadricauda* LWG002611. A similar C4-like mechanism is also described for *N. gaditana* [18].

Fatty acid biosynthesis and triacylglycerols (TAG) biosynthesis are the major biofuel metabolic pathways for lipid production (Fig. 4c). The first major step in the de novo synthesis of TAG in green algae starts in the chloroplast, where the conversion of acetyl-CoA into malonyl-CoA is catalyzed by acetyl-CoA carboxylase (ACC), the rate-limiting enzyme in TAG biosynthesis [44]. The presence of large number of gene homologs of ACC suggests the important role played by this enzyme in producing TAG precursors. Whereas, in other algae very low number of gene homologs have been observed such as 7 in *M. neglectum*, 2 in *N. gaditana* and 1 in *C. reinhardtii* [18, 21, 22]. Interestingly, only 1 gene was found for malonyl-CoA: ACP transacylase (MAT) from reference-guided assembly, whereas no gene was detected in de novo assembly. Previous reports have also indicated the low occurrence of homologous gene for MAT in other algae. Three MAT homologs were reported from *M. neglectum* [22]; whereas, a single gene was reported from *N. gaditana* [18] and *C. reinhardtii* [21]. On the other hand, 3 homologs of malonyl-CoA decarboxylase (MCD) (EC 4.1.1.9) were identified in reference-guided assembly and 4 in de novo assembly, which catalyzes the conversion of malonyl-CoA into acetyl-CoA and carbon dioxide, so to some extent, it reverses the action of ACC [45]. Thereby, the presence of lower gene numbers for MAT and presence of higher gene numbers for MCD indicates the major rate limiting step of TAG biosynthesis pathway.

Large number of gene homologs was also found for β-ketoacyl-ACP synthase (KAS), β-ketoacyl-ACP reductase (KAR), enoyl-ACP reductase (ENR) and for 3-hydroxyacyl-ACP dehydratase (HD) as compared to other reported algae. These are involved in the elongation of the acyl chains using malonyl-ACP and acetyl-CoA as substrates. Only 1 gene homolog for KAS and 1 for ENR was reported in *M. neglectum* as well as single gene homolog for HD was also observed in *N. gaditana* and C. *reinhardtii* [10, 13, 14]. These sets of enzymes are highly conserved throughout all kingdoms of life [46].

As analysed, medium-chain acyl-[acyl-carrier-protein] hydrolase is completely absent [EC 3.1.2.21], so the formation of octanoic-ACP (C8), decanoic-ACP (C10), dodecanoic-ACP (C12) is restricted. The formation of tetradecanoic-ACP is also restricted as the enzyme fatty acyl-ACP thioesterase B [EC: 3.1.2.14] is also absent. As a result there is no production of undesired fatty acids in biodiesel of carbon length C8–C14 (Fig. 1d). Whereas, 4 copies of acyl-[acyl-carrier-protein] desaturases [EC:1.14.19.2] were identified from de novo assembly and 6 from reference-guided assembly, that catalyze the formation of hexadecanoyl-ACP (C16:0-ACP), hexadecenoyl-ACP (C16:1-ACP), octadecanoyl-ACP (C18:0-ACP) and octadecenoyl-ACP (C18:1-ACP). The final termination of the fatty acid chain elongation is catalyzed by fatty-ACP thioesterases (FAT), which hydrolyse acyl-ACP into free fatty acids (FFA) [47]. In de novo assembly 8 thioesterase super family proteins and in reference-guided assembly 3 were identified. A single palmitoyl-acyl carrier protein thioesterase (FATB) gene was identified in reference assembly, which is known to possess high thioesterase activity for palmitoyl-ACP than other acyl-ACPs. Thereby, it will contribute for the formation of Palmitate (16:0) than the other fatty acids [48]. Interestingly, it correlated the presence of high amount of Palmitate (41.858% weight/weight) in *S. quadricauda* LWG002611 (Fig. 1d). Since the enzyme FAT determined the chain length, strain improvement efforts for manipulating fatty acid chain length is desired.

Free fatty acids (FFA) released in cytosol produce Acyl-CoA with coenzyme A (CoA) [47]. Acetyl-CoA thioesterases (ACOTs) [EC 3.1.2.2] which limits the formation of Acyl-CoA by hydrolyzing esters to FFA plus CoA were detected as 4 copies in both the assemblies [49] indicating a rate limiting step for elongation and desaturation in the endoplasmic reticulum (ER), which was also observed in earlier studies [50]. Glycerol-3-phosphate (G3P), the substrate used for the three sequential acylations of Acetyl-ACP to Diacylglycerol (DAG) formation reactions finally results in TAG [47]. Large numbers of gene homologs (14 in de novo assembly and 3 in reference based assembly) were identified in *S. quadricauda* LWG002611 for glycerol-3-phosphate acyltransferase (GPAT), which catalyze the formation of Lysophosphatidic acid (LPA) from Acyl-CoA. Interestingly, very few numbers of gene homologs were found for rest of the metabolic pathway of TAG biosynthesis. Only one homolog was observed for diacylglycerol acyltransferase (DGAT), indicating another rate limiting step for TAG production. Similarly, lesser numbers of DGAT homologs (3 DGAT type 1 and 2 homologs) were also observed in *M.* negelectum [22].

Triacylglycerol biosynthesis pathway within chloroplast is conserved in all members of Chlorophyceae [44]. DAG, in chloroplast, mainly serves as a precursor for photosynthetic membrane lipids, such as galactoglycerolipids, which contribute to more than 50% of the total glycerolipids under normal growth conditions [30]. Two independent studies have predicted the presence of both type-1 and type-2 DGAT in chloroplast and secretory pathway respectively [50]. Some studies have shown that they were induced by nitrogen starvation and also under sulphur, iron, phosphorus or zinc starvation [51, 52]. Other stress factors can trigger TAG accumulation, but to a lesser extent than nitrogen starvation [51]. This suggested that improvement in expression of DGAT isoforms could be one of the ways to increase TAG accumulation in algae. The presence of similar gene homologs to higher plants indicates that glycerolipid biosynthesis is a conserved pathway between lower to higher plants [53]. Detail functional investigation and transcriptome mapping are required to confirm the expression of gene families and to eliminate pseudogenes. Many success stories of gene manipulation for lipid biosynthesis pathways have been observed in higher plants [50] as compared to microalgae for biofuel production [17–20].

## Conclusion

Complete evaluation of biomass production, lipid metabolism and first whole genome sequencing revealed the potential of *S. quadricauda* LWG002611, a member of class Chlorophyceae and order Sphaeropleales for biodiesel production. It has exhibited high biomass and lipid yield when compared with other related species. The fatty acid profile was found very simple and relevant to biodiesel production. For further improvement of strain, complete genetic characterization has been carried out from genome sequencing. The genome sequence was assembled and annotated by de novo and reference-guided methods (NCBI accession no. of reference guided assembly is NNCB00000000). Housekeeping and metabolic pathway genes have been identified. Analysis of carbon fixation pathway and TAG biosynthesis pathway elucidates the targets further biotechnological improvement. The fatty acid profile of *S. quadricauda* demonstrated the presence of high amount palmitate (41.858%, weight/weight). A single palmitoyl-acyl carrier protein thioesterase gene was identified in reference assembly, which is known to produce higher amount of palmitate (16:0) and a lesser amount of other fatty acids. Molecular and morphological taxonomy provides insight into evolutionary and phylogenetic position of *S. quadricauda*. Further in-depth studies on *S. quadricauda* could establish it as the model algae for biofuel production.

## Methods

### Cultivation conditions

Various algal samples were collected from different parts of India and isolated by streaking method. All the laboratory isolates including *S. quadricauda* were cultivated in batch culture autotrophically in 1 L Bold’s basal medium (BBM) and mixotrophically using acetate as external carbon source in 1 L Tris–Acetate-Phosphate (TAP) medium in Erlenmeyer flask. They were grown in batch culture under uniform growth condition at a temperature of 27 °C ± 0.5 °C, a photoperiod of 14:10 h light/dark cycle and fluorescent illumination of 3000 lux [7]. After 25th day of culture biomass was harvested by centrifugation and dried by lyophylization. The biomass content of the cultures was measured in total weight of dry biomass in grams per litre of culture.

The growth patterns of the respective cultures were observed by measuring the optical density at 680 nm using a UV–VIS spectrophotometer (Spectrascan UV 2700, Thermo scientific). Biomass productivity was calculated by filtering 10 mL of culture during the logarithmic growth phase through pre-weighed, 0.45-μm nylon positive zeta membrane filters (Pall Corporation, USA) followed by drying at 55 °C in a hot air oven for 1–2 days till constant weight were achieved.

Biomass productivity was calculated by the formula given by Griffiths and Harrison [54]:

$${\text{Biomass}}\;{\text{productivity }}\left( {{\text{g}}\;{\text{L}}^{ - 1} \;{\text{day}}^{ - 1} } \right) = \left( {B_{2} - \, B_{1} } \right) \, / \, (t_{2} - \, t_{1} )$$

where, *B*_1_ and *B*_2_ are biomass concentration in g L^−1^ harvested from the two sampling points *t*_1_ and *t*_2_, respectively.

### Estimation of lipid content and analysis of biodiesel properties

Dry algal biomass was crushed and total lipid was estimated by adding chloroform and methanol (2:1 volume:volume) followed by heating in soxhlet apparatus for 6–7 cycles for extraction. The solvent was dried by rotary evaporator. The percentage of total lipid and lipid content was calculated by the formula described by Nag Dasgupta et al. [7]. Briefly, percentage of total lipid (Lipid%) in dry biomass was calculated by the following formula:

$${\text{Lipid}}\% = {\text{weight}}\;\left( {{\text{extracted}}\;{\text{lipid}}} \right)/{\text{weight}}\;\left( {{\text{biomass}}\;{\text{taken}}} \right) \times 100$$

The lipid content was calculated by the following formula:

$${\text{Lipid content }}\left( {{\text{mg}}\;{\text{L}}^{ - 1} } \right) \, = {\text{Lipid}}\% \times {\text{Biomass content}}$$

The lipid productivity was calculated by the formula given by Griffiths and Harrison [54]:

$${\text{Lipid productivity }}\left( {{\text{mg}}\;{\text{L}}^{ - 1} \;{\text{day}}^{ - 1} } \right) = {\text{Lipid}}\% \times {\text{Biomass productivity}}$$

The extracted lipid was refluxed for 5 h in round bottom flask at 50 °C in the presence of methanol and 2% sulphuric acid for transesterification. After removal of impurities the FAME mix was dissolved in hexane and analyzed by Gas-Chromatography (Thermo Fisher Scientific) and quantified against a standard FAME mix (Supelco, USA). Biodiesel properties of FAME were estimated from the percentage of fatty acids (weight/weight) obtained in a Gas-Chromatographic analysis using the online software “BiodieselAnalyzer© Ver. 2.2” (http://www.brteam.ir/biodieselanalyzer) [55].

### DNA isolation, draft genome sequencing and assembly

Total genomic DNA was extracted from lyophilized and crushed biomass (100 mg) using Qiagen DNAeasy Plant Mini Kit. Draft genome sequencing was performed utilizing Ion Torrent Proton NGS Platform (Bioserve, Hyderabad). Clear Reads were obtained by trimming and filtering of low quality reads and adapter sequences using CLC bio Genomic Workbench Version 8.0 and 9.0.

De novo genome assembly was done on CLC bio Genomic Workbench 9.0 with parameter Mapping Mode (Map reads back to contig), Update contigs (yes), Autometic bubble size (yes), Minimum contig length (200), Autometic word size (yes), Perform scaffolding (yes), Auto-detect paired distance (yes), Mismatch cost (1), Insertion cost (1), Deletion cost (1), Length fraction (0.7), Similarity fraction (0.6) and annotation was performed using Augustus gene prediction tool (version 3.1.0) with parameter species (chlamy2011), UTR (off), strand (both), alternatives-from-sampling (false), genemodel (partial) and BLASTx tool (Rapsearch v2.23) with parameter − *a* (fast mode (*t*/*T*: perform fast search)), − *l* (threshold of minimal alignment length (10)), − *e* (threshold of log10 (0.001) (use log10 (*E*-value)/*E*value as threshold *t*/*T*: print hits using log10 (*E*-value))), − *b* (number of database sequence to show alignments (1)), − *v* (number of database sequences to show one-line descriptions (1)), − *t* (type of query sequences (*n*/*N*:nucleotide)), − *g* (perform gap extension to speed up (*t*/*T*: perform gap extension)), − *w* (perform HSSP criteria instead of *e*value criteria (*t*/*T*: perform HSSP criteria)), − *p* (output ALL/MATCHED query reads into the alignment file (*t*/*T*: output all query reads)] [56]. Predicted genes were annotated for functional information on proteins using different bioinformatics tools such as UniProt Knowledgebase (UniProtKB), GO Pathways and PFAM enrichment analysis [57–59].

In parallel high-quality reads were processed for reference mapping and assembly using whole genome sequence of *M. neglectum* (acc. no NW_014013625.1) [22]. The FASTAQC application was applied to remove the unusual data set and CLC bio Genomic Workbench 9.0 software was used to incorporate unique features and algorithms. After extracting the consensus sequences from the mapping file, the sequences were processed in ‘Quality Assessment Tool for Genome Assemblies’ (QUAST) software for genome assembly statistics. Reference assisted genome assembly was submitted and reviewed in NCBI (Bioproject). Metabolic pathways were reconstructed by Kyoto Encyclopedia of Genes and Genomes (KEGG) (http://www.genome.jp/kegg/tool/map_pathway.html) using KO identifiers (K numbers) to individual genes in the database.

### Phylogenetic analysis and taxonomy

Phylogenetic analysis was carried out from de novo assembly and annotation of the genome sequence. The phylogenetic tree was prepared by using ‘Maximum Likelihood’ method based on the Tamura-Nei model and software MEGA5 [60].

Identification of the organism was carried out by determining the sequence of small subunit (SSU) 18S rRNA gene and morphological features. 18S rRNA gene was amplified by polymerase chain reaction (PCR) with microalgae specific forward primer (5′-GTCAGAGGTGAAATTCTTGGATTTA-3′) and reverse primer (5′-AGGGCAGGGACGTAATCAACG-3′) based on the conserved domain region of 18S rDNA [61]. The sequence (Chromous Biotech Pvt. Ltd., Bangalore) was compiled by ApE software (A plasmid Editor). The rDNA sequence was submitted to NCBI gene bank. The sequence was also blasted against the NCBI data base. The blast output was processed for generating the phylogenetic tree in Newick format [62]. The tree was redrawn using Figtree v1.4.3 software (http://tree.bio.ed.ac.uk/software/figtree) [63].

Morphological identification carried out under a light microscope (Leica DM 500) attached to Leica EC3 Camera and computerized image analysis system using monographs) [39–41]. The organism was deposited in CSIR-National Botanical Research Institute herbarium (LWG) with the accession number LWG002611.

## Additional files

**Additional file 1.** Clean read report.

**Additional file 2.** De novo assembly report.

**Additional file 3.** Reference-guided assembly report.

**Additional file 4.** De novo assembly gene ontology.

**Additional file 5.** Reference-guided assembly gene ontology.
