# Supplementary material for: Draft genome sequence and detailed characterization of biofuel production by oleaginous microalga Scenedesmus quadricauda LWG002611
Source: Biotechnol Biofuels. 2018 Nov 9;11:308. doi: 10.1186/s13068-018-1308-4 (PMC6225629; doi:10.1186/s13068-018-1308-4)

Additional file 2: S2 (de novo assembly report)

Table of contents

1. Algae_Clean_Reads_Single assembly summary report 3
    1.1 Nucleotide distribution 3
    1.2 Contig measurements 3
    1.3 Accumulated contig lengths 4
    1.4 Summary statistics 5
    1.5 Distribution of read length 5
    1.6 Distribution of matched read length 6
    1.7 Distribution of non-matched read length 6

1. Algae_Clean_Reads_Single assembly summary report

1.1 Nucleotide distribution

Nucleotide	Count	Frequency
Adenine (A)	18,702,157	18.8%
Cytosine (C)	31,096,530	31.3%
Guanine (G)	30,980,273	31.2%
Thymine (T)	18,602,060	18.7%

1.2 Contig measurements

N75	996
N50	2,112
N25	16,976
Minimum	358
Maximum	368,038
Average	1,704
Count	58,317

Total	99,381,020

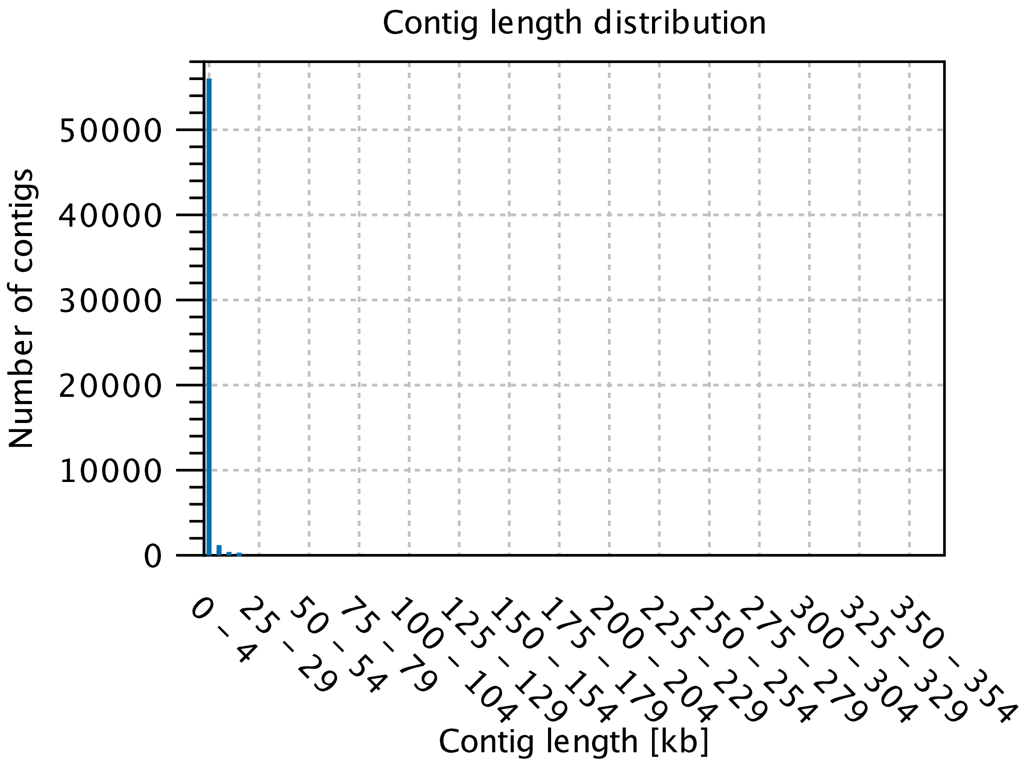

1.3 Accumulated contig lengths

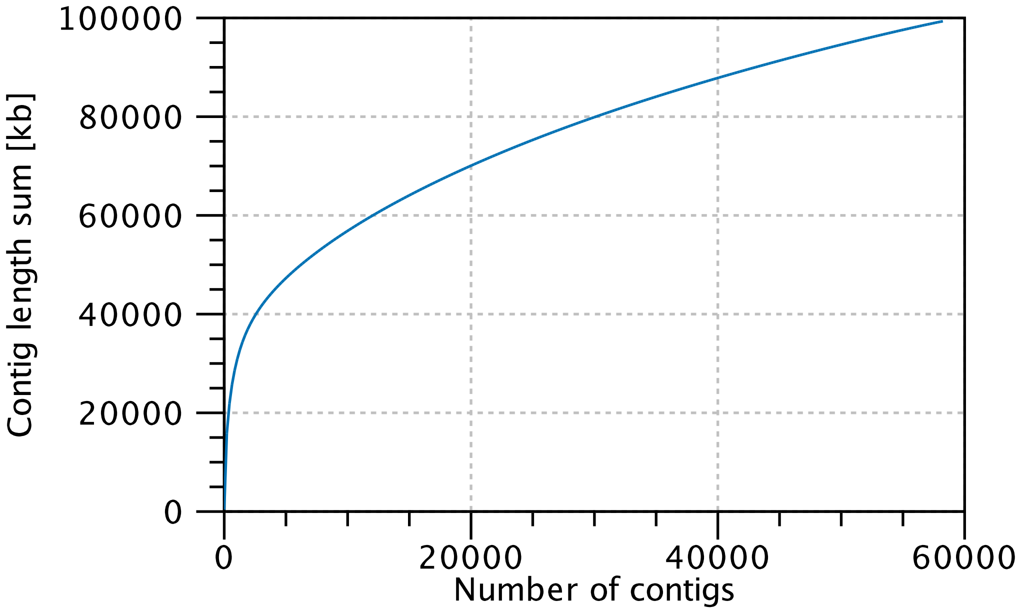

1.4 Summary statistics

	Count	Average length	Total bases
Reads	57,273,557	158.63	9,085,359,768
Matched	53,419,545	158.72	8,478,965,014
Not matched	3,854,012	157.34	606,394,754
Contigs	58,317	1,704	99,381,020

1.5 Distribution of read length

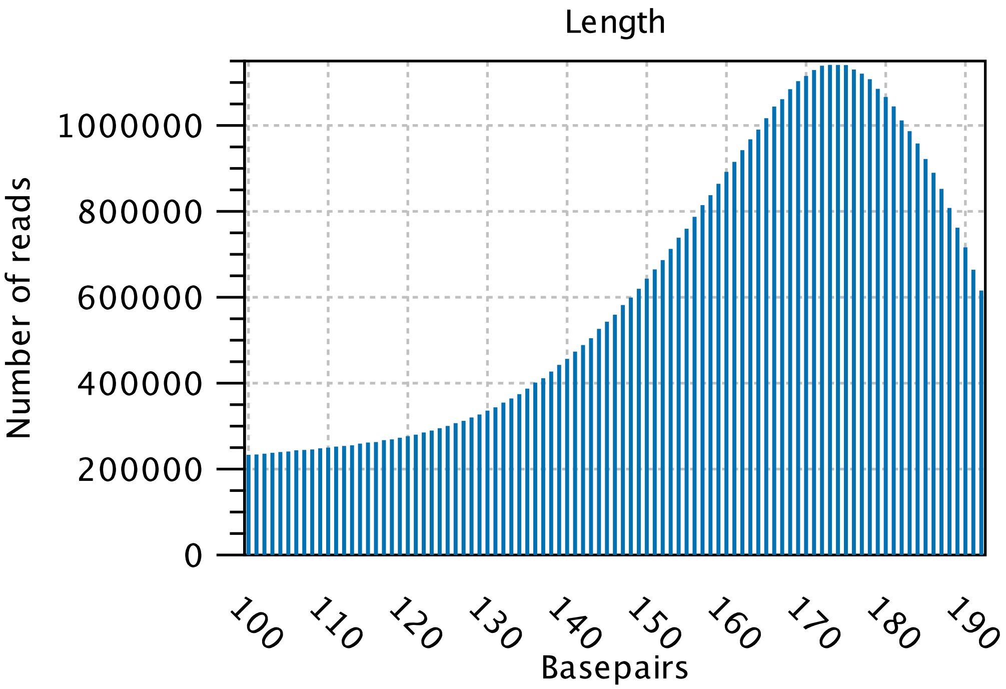

1.6 Distribution of matched read length

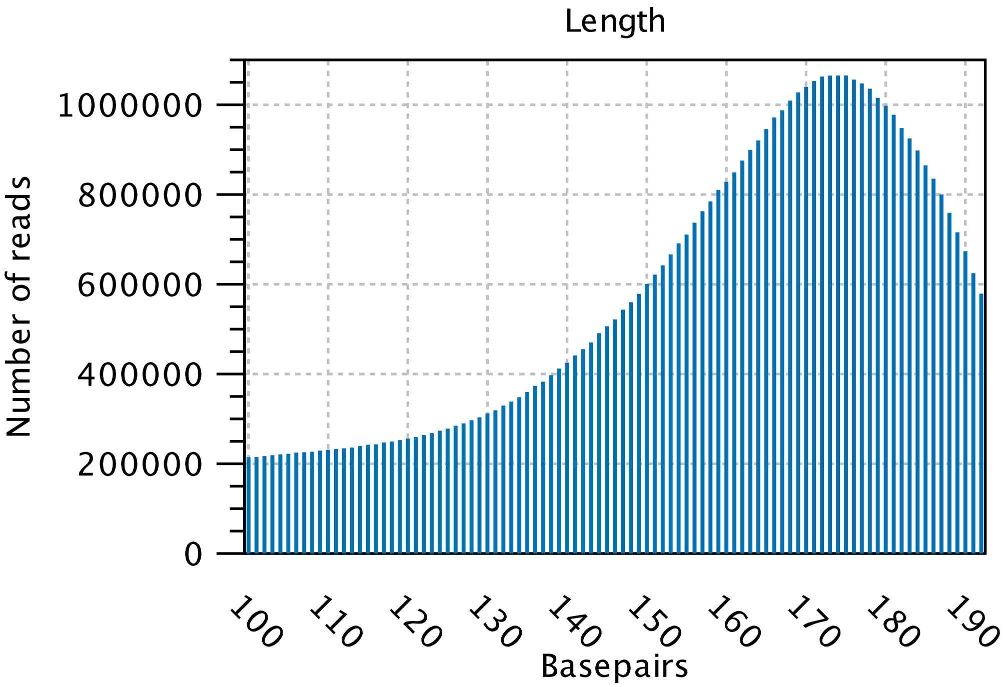

1.7 Distribution of non-matched read length

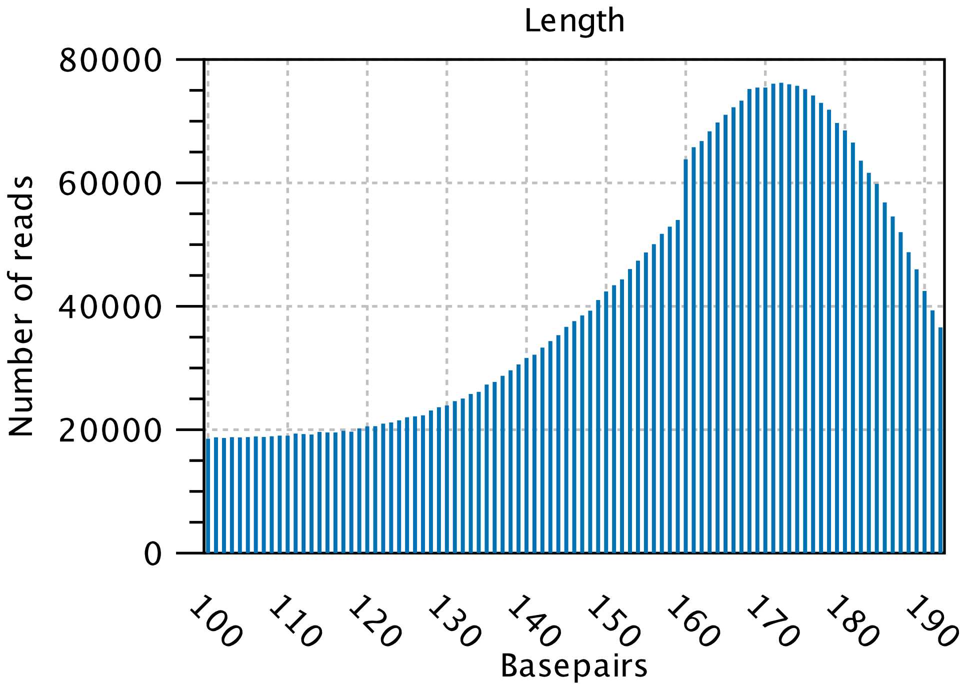

Supplement: Supplementary file 2 — Additional file 2. De novo assembly report. [file 13068_2018_1308_MOESM2_ESM.pdf]
