# Supplementary material for: Draft genome sequence and detailed characterization of biofuel production by oleaginous microalga Scenedesmus quadricauda LWG002611
Source: Biotechnol Biofuels. 2018 Nov 9;11:308. doi: 10.1186/s13068-018-1308-4 (PMC6225629; doi:10.1186/s13068-018-1308-4)
Supplement: Supplementary file 3 — Additional file 3. Reference-guided assembly report. [file 13068_2018_1308_MOESM3_ESM.pdf]

Additional file 3: S3 (Reference-guided assembly report)

Table of contents

1. Summary ... 3
2. Per-sequence analysis ... 3
    2.1 Lengths distribution ... 3
    2.2 GC-content ... 4
    2.3 Ambiguous base-content ... 4
    2.4 Quality distribution ... 5
3. Per-base analysis ... 5
    3.1 Coverage ... 6
    3.2 Nucleotide contributions ... 7
    3.3 GC-content ... 8
    3.4 Ambiguous base-content ... 8
    3.5 Quality distribution ... 9
4. Over-representation analyses ... 9
    4.1 Enriched 5mers ... 10
    4.2 Sequence duplication levels ... 11
    4.3 Duplicated sequences ... 11

1. Summary

Creation date:	Thu Jul 06 11:02:26 IST 2017
Generated by:	root
Software:	CLC Genomics Workbench 9.0
Based upon:	1 data set
R_wgs:	109,403,974 sequences
Total sequences in data set	109,403,974 sequences
Total nucleotides in data set	21,769,193,723 nucleotides

2. Per-sequence analysis

2.1 Lengths distribution

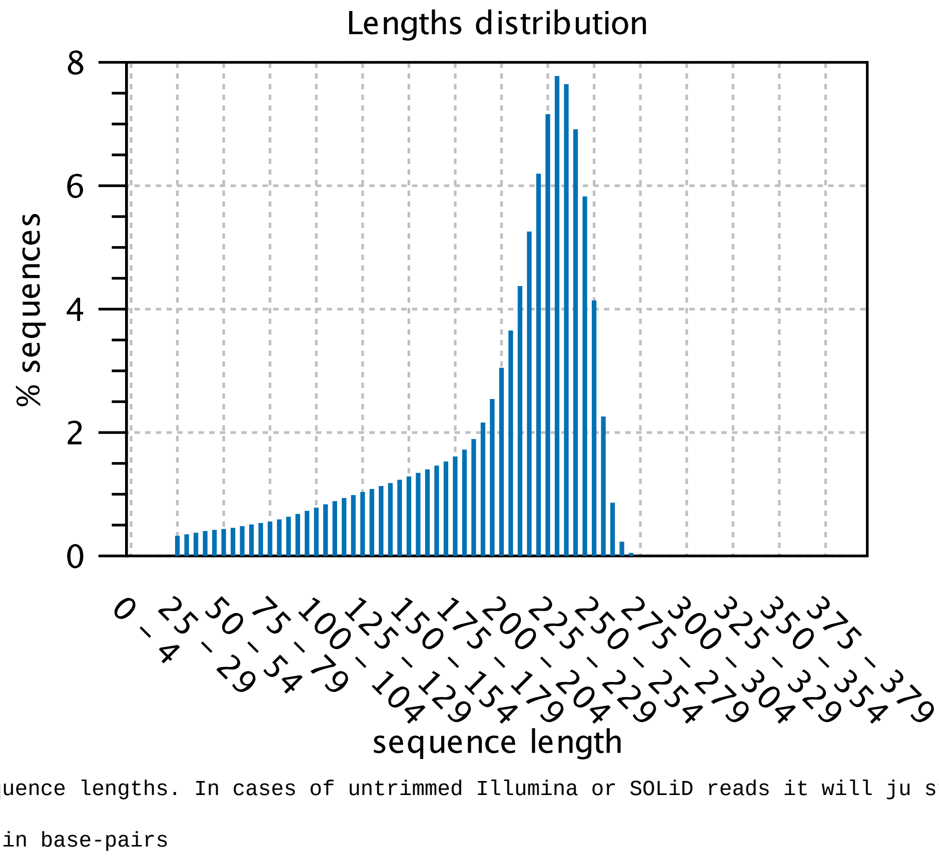


```
Distribution of sequence lengths. In cases of untrimmed Illumina or SOLiD reads it will ju st contain a single
peak.
x: sequence length in base-pairs
y: number of sequences featuring a particular length normalized to the total number of seq uences
```

2.2 GC-content

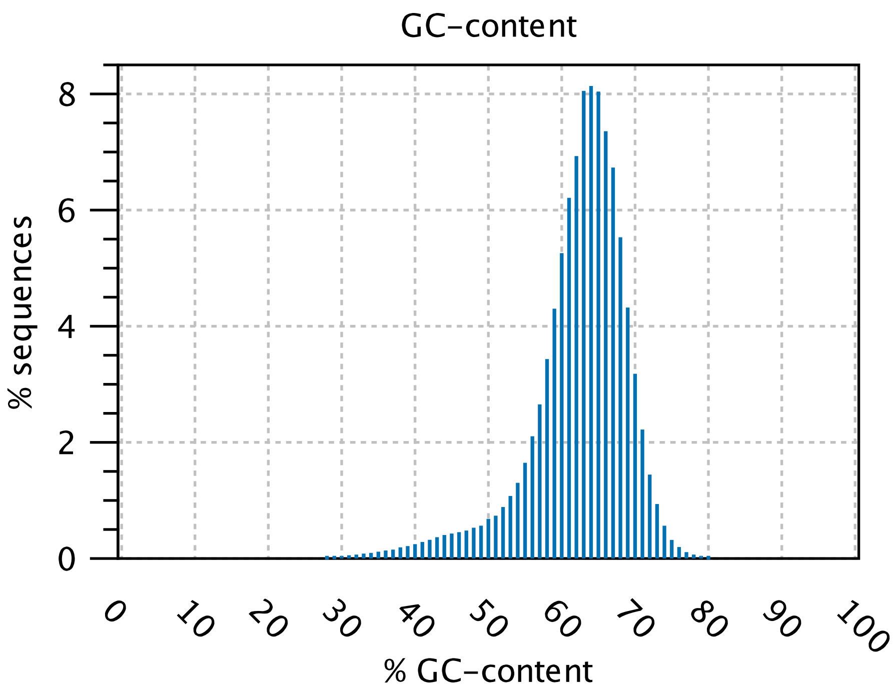

Distribution of GC-contents. The GC-content of a sequence is calculated as the number of G C-bases compared to all bases (including ambiguous bases).
x: relative GC-content of a sequence in percent
y: number of sequences featuring particular GC-percentages normalized to the total number of sequences

2.3 Ambiguous base-content

No ambiguous bases detected

2.4 Quality distribution

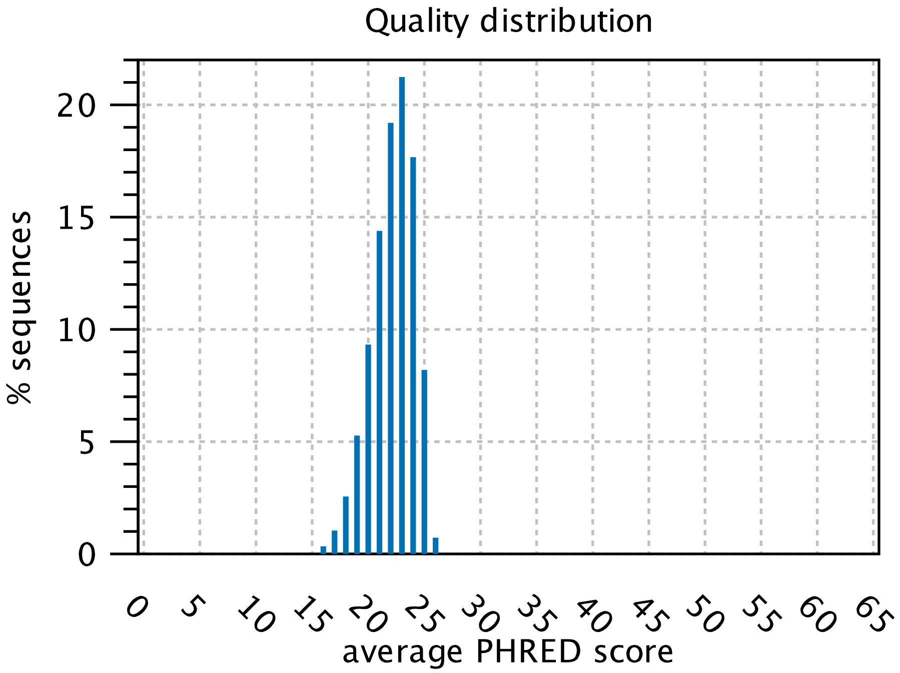

Distribution of average sequence qualitie scores. The quality of a sequence is calculated as the arithmetic mean of its base qualities.
x: PHRED-score
y: number of sequences observed at that qual. score normalized to the total number of sequ ences

3. Per-base analysis

3.1 Coverage

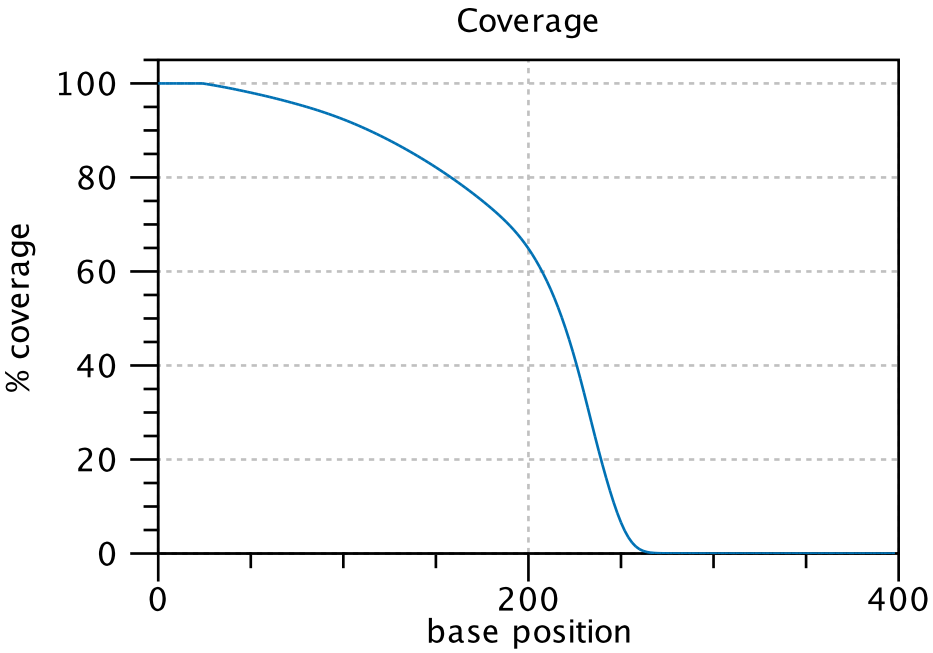

The number of sequences that support (cover) the individual base positions. In cases of un trimmed Illumina or SOLiD reads it will just contain a rectangle.
x: base position
y: number of sequences covering individual base positions normalized to the total number o f sequences

3.2 Nucleotide contributions

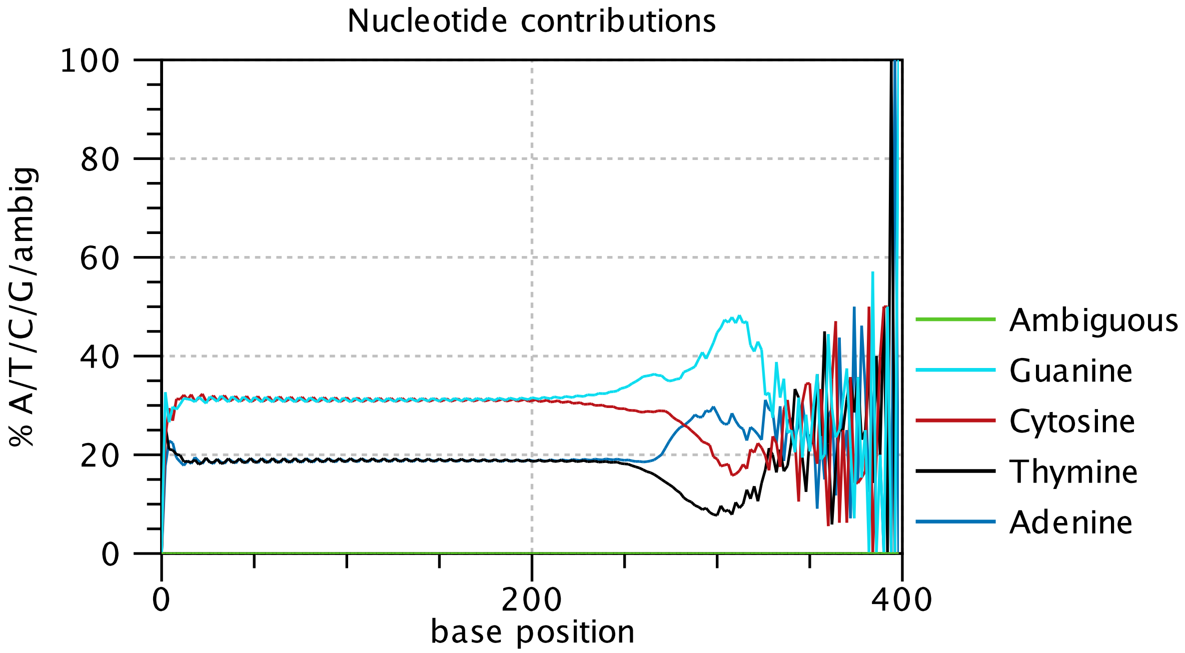

Coverages for the four DNA nucleotides and ambiguous bases.
x: base position
y: number of nucleotides observed per type normalized to the total number of nucleotides o bserved at that position

3.3 GC-content

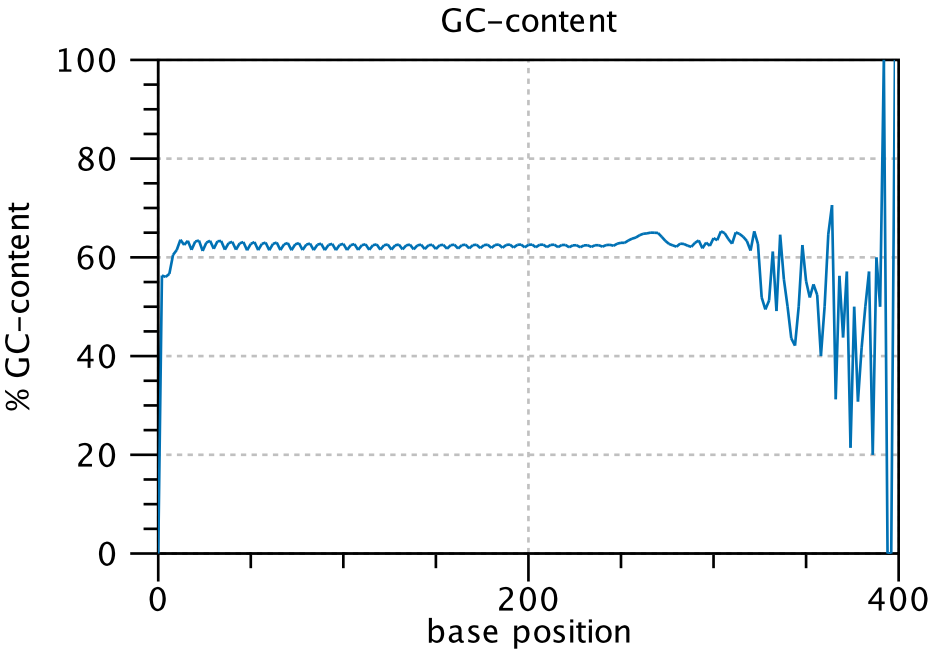

Combined coverage of G- and C-bases.
x: base position
y: number of G- and C-bases observed at current position normalized to the total number of bases observed at that position

3.4 Ambiguous base-content

No ambiguous bases detected

3.5 Quality distribution

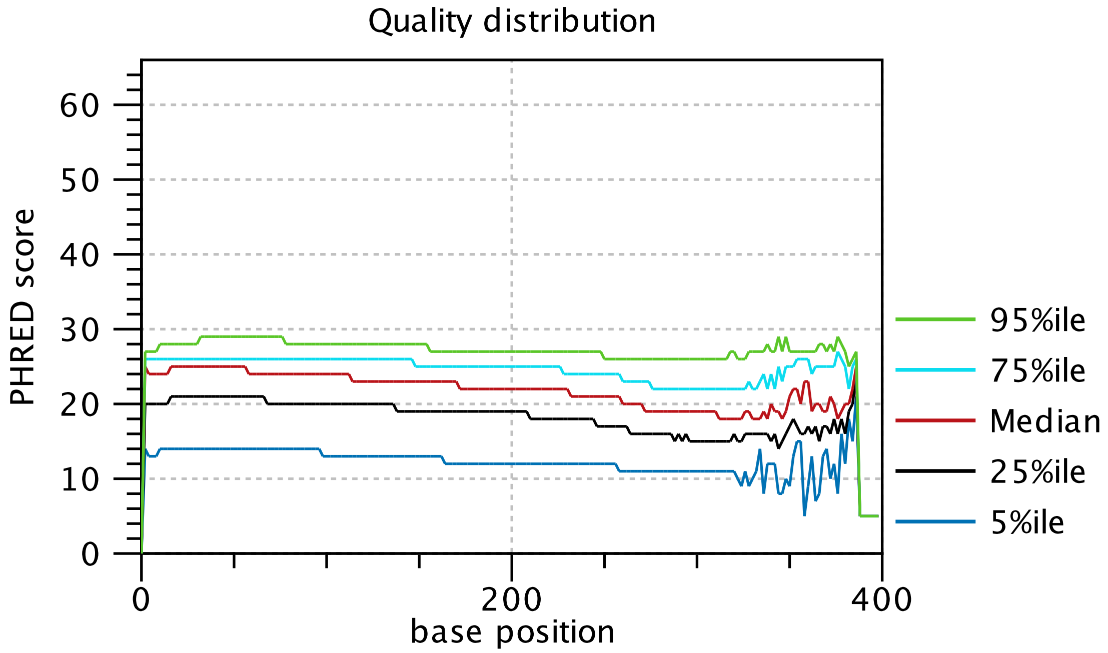


```
Base-quality distribution along the base positions.
x: base position
y: median & percentiles of quality scores observed at that base position
```

4. Over-representation analyses

4.1 Enriched 5mers

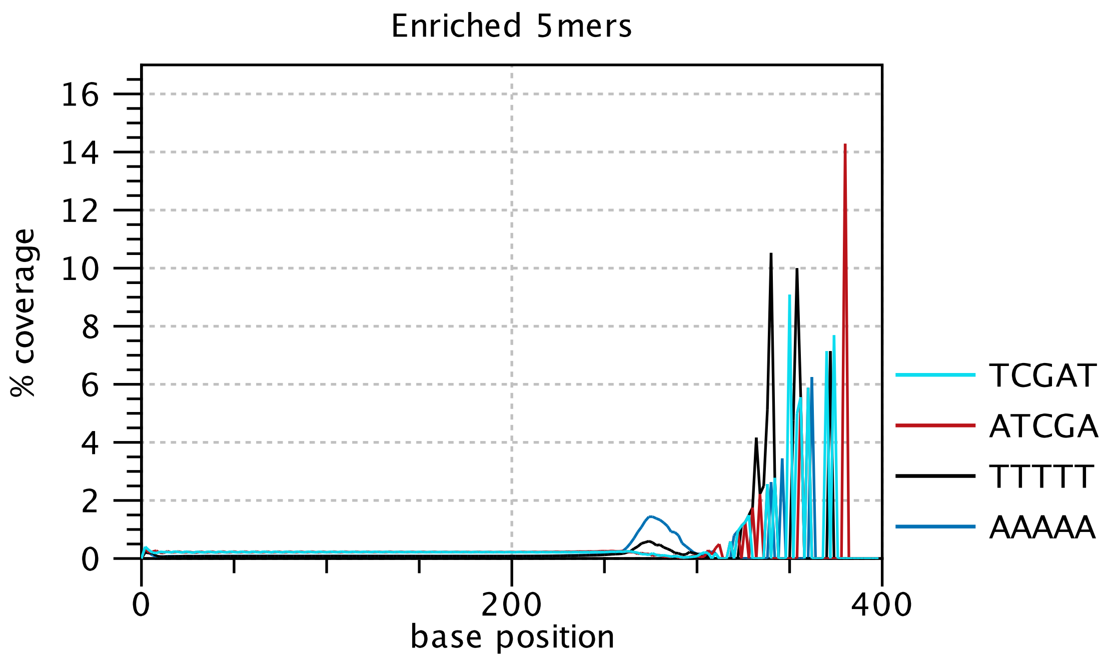

The five most-overrepresented 5mers. The over-representation of a 5mer is calculated as th e ratio of the observed and expected 5mer frequency. The expected frequency is calculated as product of the empirical nucleotide probabilities that make up the 5mer. (5mers that contain ambiguous bases are ignored)
x: base position
y: number of times a 5mer has been observed normalized to all 5mers observed at that posit ion

4.2 Sequence duplication levels

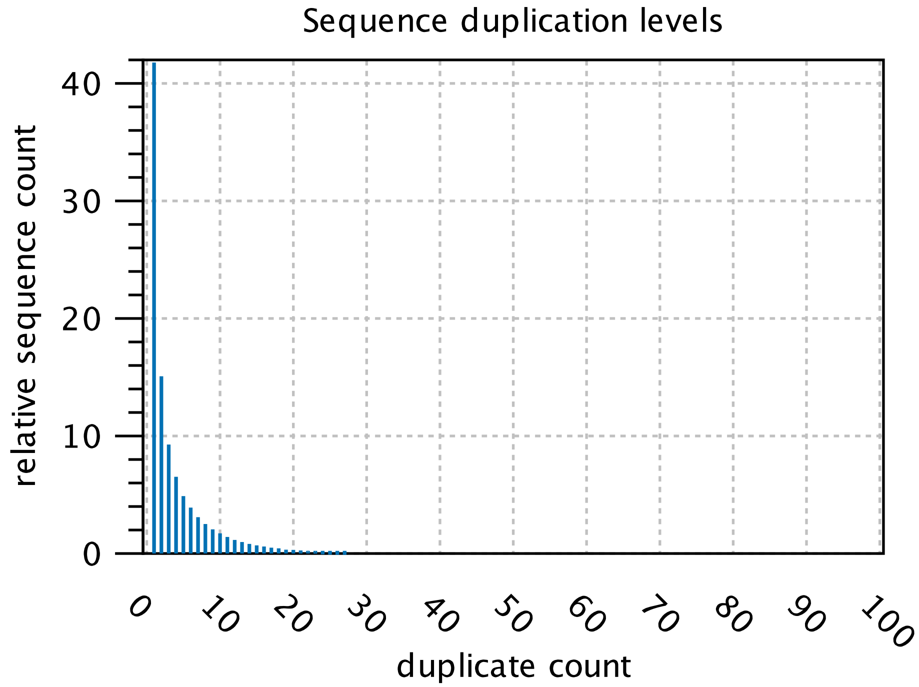

Duplication level distribution. Duplication levels are simply the count of how often a par ticular sequence has been found.
x: duplicate count
y: number of sequences that have been found that many times normalized to the number of un ique sequences

4.3 Duplicated sequences

A table of over-represented sequences is given in the supplementary report
